# High Magnetic Performance in MnGa Nanocomposite Magnets

**DOI:** 10.3390/nano14151245

**Published:** 2024-07-24

**Authors:** Ovidiu Crisan, Alina Daniela Crisan

**Affiliations:** National Institute for Materials Physics, P.O. Box MG-7, 077125 Magurele, Romania; alina.crisan@infim.ro

**Keywords:** MnGa tetragonal phase, permanent magnets, high coercivity

## Abstract

In view of their potential applicability in technology fields where magnets are required to operate at higher temperatures, the class of nanocomposite magnets with little or no rare earth (RE) content has been widely researched in the last two decades. Among these nanocomposite magnets, the subclass of magnetic binary systems exhibiting the formation of L1_0_ tetragonal phases is the most illustrious. Some of the most interesting systems are represented by the Mn-based alloys, with addition of Al, Bi, Ga, Ge. Such alloys are interesting as they are less costly than RE magnets and they show promising magnetic properties. The paper tackles the case of MnGa binary alloys with various compositions around the Mn_3_Ga stoichiometry. Four MnGa magnetic alloys, with Mn content ranging from 70 at% to 75 at% were produced using rapid solidification to form the melt. By combining structural information arising from X-ray diffractometry and transmission electron microscopy with magnetic properties determined by vibrating sample magnetometry, we are able to document the nature and properties of the structural phases formed in the alloys in their as-cast state and upon annealing, the evolution of the phase structure after annealing and its influence on the magnetic behavior of the MnGa alloys. After annealing at 400 °C and 500 °C, MnGa alloys are showing a multiple-phase microstructure, consisting of co-existing crystallites of L1_0_ and D0_22_ tetragonal phase. As a consequence of these structurally and magnetically different phases, co-existing within the microstructure, promising magnetic features are obtained, with both coercive fields and saturation magnetization exceeding values previously reported for both alloys and layers of MnGa.

## 1. Introduction

There is now a huge effort for counteracting the depletion of RE raw materials by actively searching for novel nanocomposite magnets without RE. This is particularly valid for applications where high operating temperatures are needed. It is known that due to their increased Curie temperature, the magnetic materials without RE are able to operate in media requiring higher temperatures. Several potential classes of magnetic materials with no RE in their composition were studied until now, mostly being compounds obtained in binary alloys such as FePt [1], MnAl [2] or MnBi [3]. The common ground for all these alloys is that they crystallize in certain instances into the tetragonal L1_0_ phase, a high order distortion of a cubic precursor, that was proven to exhibit good magnetic anisotropy as well as strong coercive fields [1]. The most intensive research efforts were until now devoted to L1_0_ FePt. These systems were widely studied in many different layouts, for various applications. By using chemical reduction, FePt is obtained in the shape of nanoparticles, which have good bio-compatibility for catalysis [1]. FePt was also studied in view of application for hyperthermia therapies [4], MRI diagnostics [5] and moreover for molecular recognition drug delivery [6]. Besides these applications, this system was also proposed in catalysis applications [7], as well as for sensor applications [8,9]. Due to the need of reproducibility and rigorous tailoring of sizes and degree of dispersion, the parameters for nanoparticles fabrication routes need to be strictly controlled. As such, various chemistry routes were proposed. Some of these routes, such as the thermal decomposition [10], polyol synthesis [11], the method of reverse micelles [12], microwave technologies [13], as well as self-assembly [14] and plasma technology in liquid carrier [15] and gas phase condensation [16], were proposed and found to be useful for a variety of nano-devices. Moving towards higher dimensionalities, as bulk materials, FePt alloys may operate in difficult environments: high operating temperatures and corrosive media, especially due to their impressive magnetic features: large magneto-crystalline anisotropy (10^7^ MJ/m^3^), as well as a Curie temperature that is higher than the RE-containing magnetic materials (470 °C), combined to a strong saturation magnetization (1.4 T) [17,18]. For applications requiring large amounts of magnets, the chemical synthesis methods are not appropriate, as they cannot furnish sufficient quantities, relevant for industrial use. In that aspect, bulk synthesis technologies, such as ultra-rapid solidification or melt spinning may be more suitable for RE-free alloys [19]. A previous report [19] proved that FePt-based alloys can be obtained directly in the L1_0_ phase if the alloy was cast as a melt spun ribbon. Moreover, such a synthesis route was suitable for achieving the L1_0_ phase without thermal annealing, as is the case for chemically prepared nanoparticles. Such a synthesis route was, for instance, suitable for another system where the L1_0_ phase can be formed, such as the MnGa alloy. This is a particular case, the MnGa binary system, as it exhibits a rather complex phase structure, especially when it comes to the Mn-rich interval in the corresponding phase diagram. As such, a synthesis procedure able to obtain the desired L1_0_ phase, with a higher probability than other thermodynamically competing phases—the case of the melt spinning method—appears rather convenient for attempting to produce L1_0_ MnGa alloys.

The phase diagram of the binary system MnGa [20] shows a plethora of interesting magnetic phases, around the point of 70–75 at% Mn, where no less than three crystal structures may be formed and co-exist. Hexagonal D0_19_ phase Mn_3_Ga behaves as an antiferromagnet with non-collinear structures. It has been studied in regards to complex phenomenology such as exchange bias, exchange coupling at the interfaces as well as in materials showing giant magnetoresistance effect [21]. Another structure is the tetragonal D0_22_ phase Mn_3_Ga. This one is a ferrimagnet. It has been usually investigated for instance for switching via the spin torque effect [22]. Another potential application of the Mn_3_Ga D0_22_ phase has been identified in the field of spintronic devices which show THz emission [23]. The third concurrent phase in the vicinity of the 75:25 Mn to Ga relative abundance in the phase diagram is the tetragonal L1_0_ MnGa. This one is a ferromagnet with high magnetic anisotropy, exhibits high coercive field and large magnetization [24,25]. Such unusual kind of magnetism was discussed in [26] and linked to the electronic structure of the Mn atoms. As the *d* levels of Mn atoms are half-filled with electrons, this can support a wide range of potential chemical environments [26] and for this reason their electronic properties vary in a quite narrow relative atomic range, around the vicinity of 75 at% Mn. As an example, a structural phase transition involving formation of tetragonal L1_0_ in MnGa was studied in ion irradiated binary films [27]. In this reference, it is argued that the microstructure obtained after irradiation consists of L1_0_ ordered crystals embedded into a cubic disordered MnGa residual matrix. Moreover, while the coercive field has increased, the magnetic anisotropy was not at all influenced by the irradiation process.

Actual occurrence of the L1_0_ tetragonal structure was found also in alloyed layers of Mn-Ga with a content of 52 at% up to a content of 60 at% Mn [28]. In this instance, the magnetization component along the layer plane was determined to be lower as the Mn concentration increased. This phenomenon was considered to be due to some competing interactions, imposed by the non-collinear structure, at the interface with the semiconducting substrate. Other works [29] used thin films of the binary MnGa alloys, deposited onto a Co-Ga buffer substrate that produced a strong coercive field of about 7kOe as well as significant magnetic anisotropy.

It is worth noticing that MnGa layers were proven to be useful for making devices. For instance, Tsunegi et al. [30] proved occurrence of the spin torque diode effect in tri-layers containing MnGa shaped as magnetic tunnel junctions. A second usefulness of L1_0_-MnGa films was mentioned by Zhu et al. [31] where a very high anomalous Hall effect was found in systems with coercive fields of about 12 kOe. Epitaxial MnGa layers were shown to exhibit coercive fields of about 10 kOe [32]. As an important magnetic parameter for permanent magnets, the coercive field as well as the energy product BH_max_ is the most important figure of merit in potential applications. For all of the above reasons, to optimize the material phase structure and phase compositions in view of obtaining enhanced coercive fields constitutes main drive of research into advanced magnetic materials. For correct estimation of coercive fields and magnetic moments, leading to correct determination of the specific magnetization, one of the most suitable techniques, used also for determining magnetic performances in other multiple phased materials [33,34], is the vibrating sample magnetometry. Some of the highest ever reported values in bulk alloys of MnGa (13.5 kOe) were found only after thermal annealing in the applied magnetic field [25].

This paper presents a study of MnGa binary alloys, with compositions in the vicinity of 75:25 at% Mn to Ga. It is reported that formation of the L1_0_ and D0_22_ tetragonal phases occurs from the hexagonal precursor in Mn_3_Ga, Mn-rich, alloys, fabricated via the melt spinning method. Not only that, high magnetic parameters, coercive fields and saturation magnetization, is evidenced for annealed alloys. This proves that among other L1_0_-based magnetic materials, the systems based on MnGa are promising as performant and cost-effective magnets in several industrial applications.

## 2. Materials and Methods

For optimizing magnetic features which are underlined by the half filling of the *d* shell of Mn atoms, we chose the MnGa composition of the alloy closer to the Mn_3_Ga ratio. Taking this into account, the initial nominal composition for the two alloys was 73:27 and 75:25 at% Mn to Ga ratio. The procedure of synthesis, ultra-rapid solidification from the melt, involves a Buehler met spinning facility (Buehler Melt Spinner MSP 10 from Edmund Buehler GmbH, Bodelshausen, Germany). For the synthesis of the initial alloy, metal flakes of Mn and Ga of quite high purity 99.99% from Merck (Merck KGaA, Darmstadt, Germany) were used. Pre-alloying of the precursor is performed by melting in an arc oven, in controlled argon atmosphere, with low pressure of 10^−1^ Torr. The obtained pre-alloy is then re-melted no less than three times, in the same oven. This is to ensure a high degree of homogeneity of the alloy and a complete alloying of the initial elements. In the next step of the synthesis procedure, a quantity of 5 g of pre-alloy is introduced into the quartz crucible mounted inside of the melt spinner. The crucible is mounted on top of the rotating wheel inside of the melt spinner chamber. It is also inductively heated for bringing the pre-alloy to the molten state. There is a 3 mm nozzle at the bottom of the crucible in close vicinity to the rotating copper wheel. By the time the pre-alloy is completely melt inside the crucible, an argon over pressure of 50 kPa is inserted inside the crucible. The immediate consequence is that the melt is completely and instantly purged onto the surface of the rotating copper wheel. The frequency of rotation for the copper wheel is set to roughly 1150 rot/min, which is equivalent to peripheral velocity of about 26 m/s. In contact with the fast-rotating copper wheel, the melt solidifies almost instantly with a cooling rate of about 10^5^ K/min, giving rise to continuous ribbons, more than 5 decimeters long, 2.5 mm wide and about 45 microns thick.

The as-cast ribbons are furthermore thermally annealed, in order to promote formation of tetragonal L1_0_ and/or D0_22_ phases. For this purpose, an isothermal annealing procedure of 1 h duration at various temperature values between 400 °C and 700 °C takes place. The annealing procedure is performed in a controlled atmosphere resistive oven, in low vacuum of 10^−3^ Torr.

For accurate measuring of the actual composition of the two as-cast samples, the technique of energy-dispersive X-ray spectroscopy (EDX or EDS) is used. For this purpose, we are using the EDX external module of the Evo 50 XVP from Carl Zeiss NTS (Carl Zeiss Nano Technology Systems GmbH, Oberkochen, Germany) scanning electron microscope.

Electron microscopy imaging using transmission electron microscopy in its high-resolution mode, accompanied by electron diffraction images of the reciprocal space are used for obtaining the actual microstructure of the as-cast and annealed ribbons and was performed using a JEM-ARM200F high resolution microscope from JEOL Ltd. Europe, Zaventem, Belgium. This microscope, operating at 300 kV acceleration voltage, is capable of achieving 1.7 Angstroms lateral resolution.

The structural characterization was performed through X-ray diffractometry using a D8 Advance diffractometer (Bruker AXS GmbH, Karlsruhe, Germany). X-ray diffractograms were obtained in the powder diffraction mode, in ambient conditions, under a θ–2θ incidence geometry. The angular interval of analysis was between 20° and 90° (in 2θ), and the X-ray beam used was the Cu Kα radiation with a wavelength λ = 0.154 nm. For full-profile analysis of the obtained diffractograms, a Rietveld-type refinement analysis was performed on all of the obtained XRD patterns. The analysis involves the use of MAUD (Materials Analysis Using Diffraction) software (MAUD version 2.99, University of Trento, Italy).

All the magnetic measurements, such as hysteresis loops and specific magnetization measurements were performed utilizing the vibrating sample magnetometry module of a MPMS (Magnetic Properties Measurement System) from Quantum Design (Quantum Design Europe GmbH, Darmstadt, Germany). This MPMS device is extremely sensitive, being capable of detecting magnetic moments as low as 10^−8^ emu. It provides up to 10^−11^ A m^2^ resolution and is capable of furnishing up to a 12 T applied magnetic field between a 2 and 400 K temperature range. The magnetic determinations were carried out on both as-cast as well as annealed samples in an applied field of up to 12 T, which was set parallel to the ribbons plane. All measurements were performed at 300 K.

## 3. Results

### 3.1. Phase Structure—X-ray Diffraction

All the synthesized ribbon samples have been analyzed using energy-dispersive X-ray spectroscopy (EDX). For all the four different MnGa alloys, with various stoichiometries, it has been revealed that their real composition is quite close to the nominal, initial stoichiometry. Table 1 presents the initial stoichiometry of the as-cast alloys as well as the measured composition using EDX. It has been seen that overall, there is a good agreement between nominal and real composition, the discrepancies being situated within a 1% error range. This accuracy is encouraging taking into account that usually in the out-of-equilibrium melt spinning fabrication procedure, as the melt is purged from the crucible through the nozzle onto the fast-rotating wheel, there could be inherent losses of material. The fact that our measured composition is similar to the nominal one, within 1% accuracy proves the good homogeneity of the melt and also speaks about the good reproducibility of the fabrication technique. This is especially important in the case of MnGa binary alloys where the structure, phase composition and consequently the magnetism of the obtained phases are highly sensitive to the slightest changes in the relative abundance of Mn and Ga in the binary systems.

As stated in the previous section, for obtaining the desired microstructure, with good magnetic properties, a thermal annealing procedure was undertaken for both samples. For the annealed samples and their as-cast counterparts, structural analysis was performed both by XRD and by TEM. During TEM analysis, also electron diffraction patterns (EDP) were recorded, giving thus indications about the microstructure. These EDP results were then compared and interpreted in view of XRD results, for a more complete assessment of the phase structure and structural parameters.

**Figure 1:**
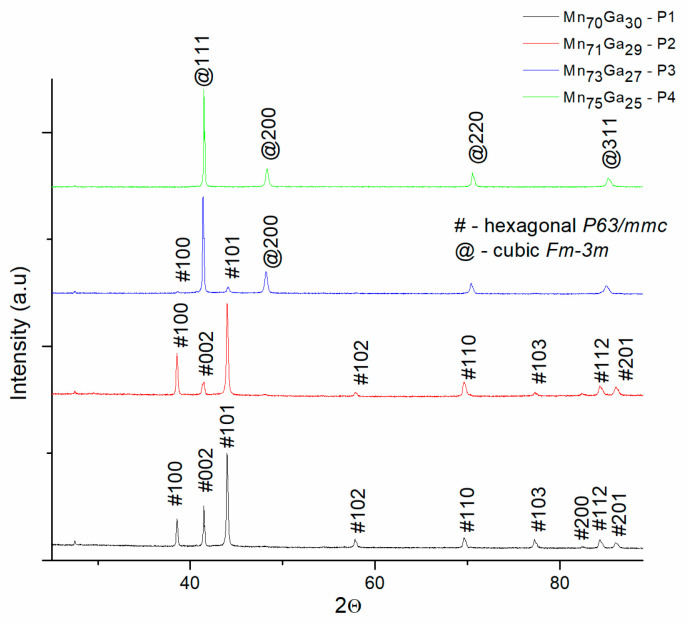
XRD patterns of all the as-cast samples, drawn from top to bottom in decreasing order of the Ga content. # indicates the *hkl* reflections of D0_19_ Mn_3_Ga hexagonal phase. @ indicates the *hkl* reflections of cubic Mn_3_Ga phase.

Figure 1 shows the XRD patterns of all the as-cast samples. The diffractograms exhibit sharp Bragg peaks, illustrating the strong crystallinity of the MnGa samples in their as-cast state. The peak structure is, however, different, which is surprising, taking into account that there are only small variations in their composition. All the observed Bragg lines have been identified by the full-profile analysis, provided by MAUD software. Previously published papers [20,21] on MnGa alloys propose that, for concentrations between 70 and 75 at% Mn ratio the alloy crystallizes in the hexagonal D0_19_ Mn_3_Ga phase. According to the phase diagram published in [20], it is one of the most stable on the whole range of compositions. For these reasons, the hexagonal D0_19_ Mn_3_Ga phase was expected to be found in the as-cast state of MnGa alloys for the range of Mn content 70 to 75 at%.

We have indeed found after full-profile analysis and identification of the Bragg peaks that for P1 and P2 samples, with Mn content 70 and 71 at%, the diffraction lines belonging unambiguously to the D0_19_ Mn_3_Ga is hexagonal, identified by matching the observed Bragg peak positions to the theoretical lines of this phase as revealed in its ICDD file 04-004-2330. It is worthwhile noticing that D0_19_ Mn_3_Ga with a Mg_3_Cd-type structure belongs to the space group is *P*6_3_/*mmc*, has hexagonal symmetry and the lattice constants, as determined from the full-profile analysis are *a* = *b* = 5.696 Å, *c* = 4.541 Å. In the D0_19_ Mn_3_Ga crystal structure, Mn atoms are disposed within a kagome-type triangular Bravais lattice in the basal planes. These planes are stacked along the *c* axis, with Ga atoms situated in the middle of Mn atoms hexagonal arrangements [21].

The situation is, however, completely different for the P3 and P4 samples, with Mn content of 73 to 75 at%. It can be seen that in the diffractogram of the P3 sample, the Bragg lines of the D0_19_ Mn_3_Ga are mostly disappearing, giving rise to a different peak structure. Only (100) and (101) *hkl* reflections of the hexagonal phase are visible with a much-reduced intensity. The main observed diffraction lines belong to the cubic Mn_3_Ga phase, as it can be seen in the indexation in Figure 1. In the diffractogram of sample P3, it can be seen, however, that there is still reminiscence of the main Bragg peak of the D0_19_ Mn_3_Ga, the (101) reflection. It is concluded thus that within this narrow range of composition, 70 to 75 at% Mn, there could be a completely different structure formed for the binary alloys. We show that, for a Mn content from 70 to 71 at%, the compound crystallizes in the D0_19_ Mn_3_Ga hexagonal structure, while from 73 to 75 at% it crystallizes in the cubic Mn_3_Ga phase structure. The finding is consistent with recent analysis of [35] where it was shown that in Mn_3_Ga with composition close to 75:25 Mn to Ga, there occur a shear-induced, temperature-prompted, back-and-forth transformation between the cubic and hexagonal structures. Moreover, when annealing at 400 °C, two different transformation pathways are identified [36], both terminating in tetragonal Mn_3_Ga as the equilibrium phase. This cited study though [36] does not investigate various compositions, around the 75:25 point, as in our report; therefore, their findings regarding the final structure obtained after annealing are only valid for a binary alloy composition at the 75:25 point in the MnGa phase diagram.

**Figure 2:**
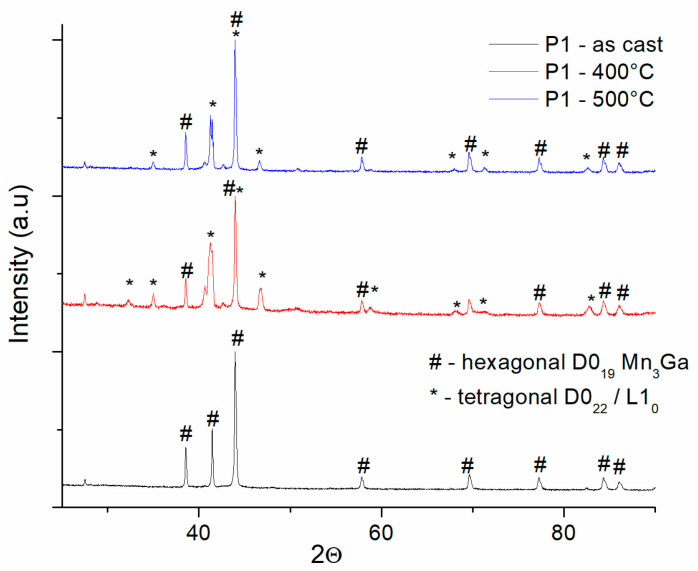
Comparative XRD diffractograms of as-cast and annealed P1 samples.

**Figure 3:**
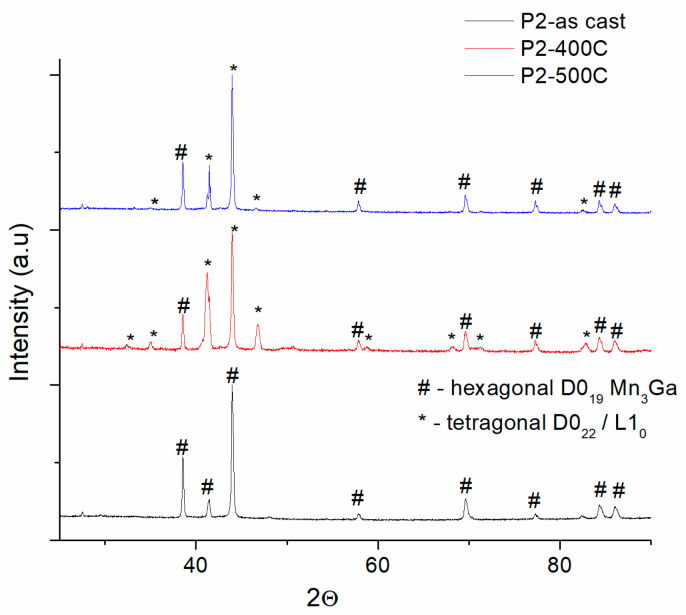
Comparative XRD diffractograms of as-cast and annealed P2 samples.

**Figure 4:**
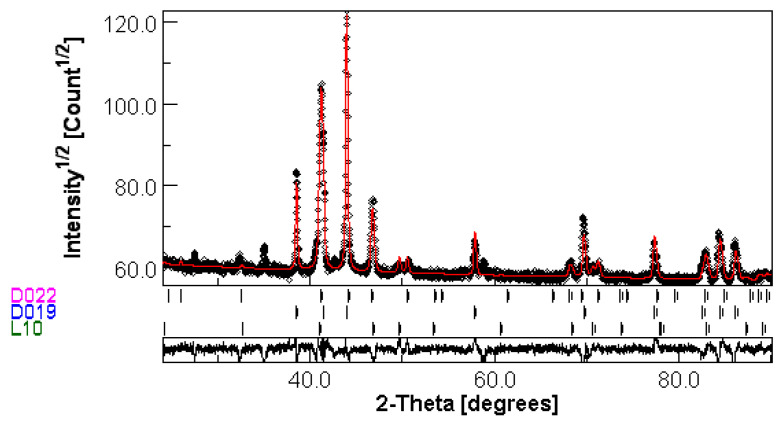
The MAUD fitting of the P2 sample annealed at 400 °C with a model consisting of D0_19_ Mn_3_Ga hexagonal, D0_22_ Mn_3_Ga tetragonal as well as the L1_0_ Mn_2_Ga tetragonal phases. Black symbols: experimental data; Red line: numerical fitting data.

**Figure 5:**
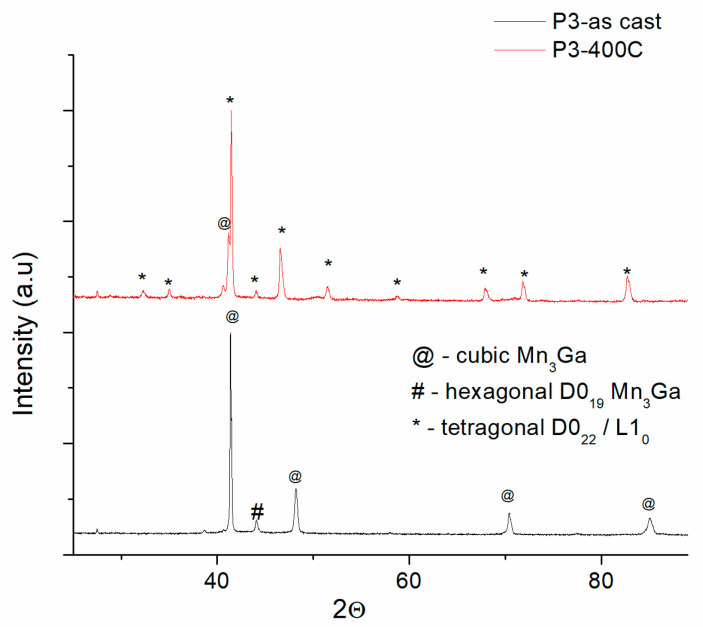
Comparative XRD diffractograms of as-cast and annealed P3 samples.

**Figure 6:**
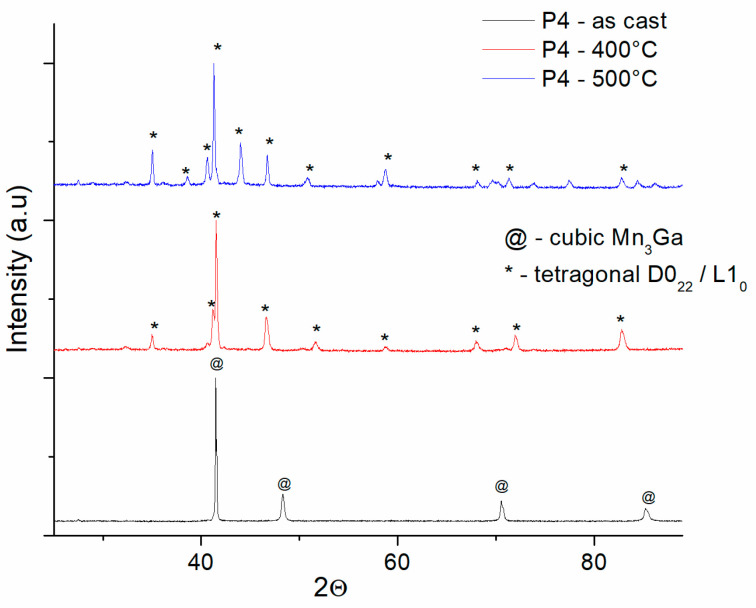
X-ray diffraction patterns of the P4 samples, as-cast and annealed at 400 °C and 500 °C.

In order to induce enhancement of magnetic properties, we have undertaken several annealing procedures, as stated in the Section 2. Namely, we have proceeded to thermally anneal for 1 h at 400 °C and 500 °C all the four samples with concentrations as listed in Table 1. We have furthermore checked the structure of the annealed samples via XRD and TEM. In Figure 2, Figure 3, Figure 4, Figure 5 and Figure 6, we display comparatively the diffractograms of as-cast and annealed samples. In this way, we can observe differences in phase structure that occur in the samples after annealing.

Figure 2 shows the XRD data of as-cast and annealed P1 samples. The annealed samples have quite different image compared to the as-cast sample. While in the as-cast state, the diffraction lines marked with # are indexed as belonging to the D0_19_ hexagonal Mn_3_Ga phase, making the as-cast state a single-phase structure, the annealed samples show, besides the peaks of the D0_19_ hexagonal phase, occurrence of additional Bragg peaks, marked with *, diffraction lines that were indexed as belonging to the tetragonal phases D0_22_/L1_0_ MnGa. Almost similar features are encountered in the case of the P2 samples (Figure 3). Here, for the P2 samples annealed at 400 °C and 500 °C, the occurrence of the tetragonal D0_22_/L1_0_ phase accompanying the co-existing hexagonal D0_19_ Mn_3_Ga phase, is observable in a larger amount than in the case of the P1 sample annealed at 400 °C, as revealed by the higher peak intensities of the correspondent Bragg lines.

The assignation of additional Bragg lines, of the newly formed *hkl* reflections that manifested in the diffractograms after annealing to the tetragonal D0_22_/L1_0_ phases, need some additional comments. In his seminal review paper [24], Mike Coey demonstrated that Mn_3−x_Ga represents from a metallurgical point of view, a continuous solid solution of tetragonal symmetry, over the whole range of variation of 0 < x < 1, respectively, from Mn_3_Ga (D0_22_) to Mn_2_Ga (L1_0_), the difference between these two tetragonal variants being the type of the lattice, primitive (*P*) for the L1_0_, respectively, and body centered (*I*) for the D0_22_ variant. The D0_22_ is in fact a largely distorted variant of the cubic Heusler L2_1_ structure, of formula XYZ_2_, where X, Y and the two Z atoms are situated on all four corners of intertwined face-centered cubic sublattices [24]. As a matter of fact, the D0_22_ structure has high resemblances to the tetragonal L1_0_, which has alternate ordered layers of only X or only Y atoms and two Z atoms, where X = Ga and Y, Z = Mn. In fact, the L1_0_ structure is obtained from the D0_22_ structure, when X and Y atoms are of the same species (Ga). Whilst both phases are tetragonal, the space group of the D0_22_ phase is *I*4/*mmm* and is observed for the Mn content between 63 and 81%, its counterpart L1_0_ tetragonal phase crystallizes in a structure having *P*4/*mmm* space group and occurs in binary MnGa alloys for a Mn content between 50 and 70% [37]. Following then the reasoning from [37] as well as observing the MnGa phase diagram [20], we can safely assume that there is a range of concentration for Mn where these two tetragonal phases, L1_0_ and D0_22_, could co-exist. Their distinctive signature in XRD patterns in the low diffraction angle part of the diffractograms is the appearance of the so-called superlattice peaks, the (001) and (110) *hkl* reflections. In what concerns the lattice parameters, *a* parameter is almost the same for the two tetragonal phases but the *c* parameter of the D0_22_ is actually twice the *c* parameter of the L1_0_ phase. This is the main difference in the estimation of whether a tetragonal phase observed in XRD is L1_0_ or D0_22_, since the angular position of the two main superlattice peaks, as shown by Niida et al. [25], the (001) and (110) Bragg reflections, are very similar.

By using the MAUD analysis, we have been able to identify, on the patterns recorded for the P1 samples annealed at 400 °C and 500 °C, the superlattice peaks (001), (011), (101) and (110) belonging to the tetragonal MnGa phases. It is, however, rather difficult to distinguish between the two tetragonal phases by only observing the XRD pattern, since the angular position of the corresponding peaks are very similar. In many published works, similar XRD patterns are said to belong either to the D0_22_ [22] or L1_0_ [26] phase structure, but without any quantitative additional proof. The only indication that a given set of superlattice peaks belong to either of the two D0_22_ and L1_0_ tetragonal MnGa phases is considered to be furnished by the Mn content. When the Mn content is closer to the stoichiometry of Mn_2_Ga (around 66 at% Mn), it is assumed that the tetragonal phase is L1_0_. If the Mn content is closer to Mn_3_Ga (around 75 at% Mn), it is assumed that the tetragonal phase is D0_22_. In our case, we undertook a full-profile analysis of the XRD patterns of all the annealed samples by using MAUD software. An example of the full-profile fitting of the X-ray diffractogram of the P2 sample annealed at 400 °C is given in Figure 4. This diffractogram, as well as the one for the P2 sample annealed at 500 °C were accurately fitted with a numerical model which considers, apart the (still) existing hexagonal D0_19_ Mn_3_Ga phase, the tetragonal D0_22_ Mn_3_Ga phase, *I*4/*mmm* space group (ICDD PDF file: 04-015-2490) as well as the tetragonal L1_0_ Mn_2_Ga phase, *P*4/*mmm* space group (ICDD PDF file: 03-065-6327). In Figure 4, the numerical fitting with the model described above is shown by the red line while experimental data are shown in black dots. It is clearly seen that the model with one hexagonal phase and two tetragonal ones fits very well the experimental data.

The lattice parameters of the two phases are listed in the Table 2:

It can be seen that while the lattice parameters of the two tetragonal phases do not vary much upon annealing, as the values for the P2 sample annealed at 400 °C do not differ much from the values for the P2 sample annealed at 500 °C, it is quite obvious that the phase transformation from the hexagonal to the tetragonal phases, induced by annealing, give rise to a higher amount of magnetic phases, as the annealing temperature increases. Whereas for an annealing at 400 °C, the total amount of tetragonal phases is 53%, for the annealing at 500 °C, this total amount increases to 59%. This result will be confirmed also when comparing the magnetic properties of the two annealed samples in the next section.

Such model of fitting has been applied also to the other XRD patterns for the samples P3 and P4. The only exception is that in the case of samples with 73 to 75 at.% Mn, the as-cast precursor phase is not the hexagonal D0_19_ Mn_3_Ga phase but the face centered cubic Mn3Ga, space group *Fm-3m*. Figure 5 and Figure 6 present the comparative depiction of the XRD patterns of the P3 and P4 samples, as-cast and annealed at 400 °C and 500 °C.

In the case of the P3 sample, having the composition Mn_73_Ga_27_, we have observed that the as-cast state mostly consists of the cubic Mn_3_Ga phase; however, a small Bragg peak, belonging to the main *hkl* reflection of the hexagonal phase, the (101) peak, is still visible, so it may be said that there is an intermediate composition where in the as-cast state, both precursor phases, the D0_19_ hexagonal and the cubic Mn_3_Ga may co-exist. However, as the intensity is very low, one may safely assume that the P3 sample has in its vast majority a cubic structure in its as-cast state. The P3 sample annealed at 400 °C, shows, however, many additional Bragg peaks of the tetragonal D0_22_/L1_0_ phases, while the peaks belonging to the cubic phase are mostly disappearing. Only the main Bragg reflection (111) of the cubic phase is observable as a shoulder to the very intense, main Bragg peak (111) of the tetragonal D0_22_ phase.

The situation is quite similar also in the case of the P4 samples, as-cast and annealed (Figure 6). Here, the as-cast state consists of a cubic Mn_3_Ga phase, as it can be seen that only the Bragg reflections of the cubic Mn_3_Ga phase, space group *Fm-3m* (ICDD PDF file: 04-001-3054) were identified by MAUD fitting. Upon annealing though, the cubic structure is not seen anymore, with the annealing at 400 °C and 500 °C causing the almost complete structural phase transformation from cubic to tetragonal D0_22_/L1_0_ phases. For both annealed P4 samples, the temperature of annealing is sufficient to transform almost completely the cubic phase into the tetragonal phases, with visible consequences also in the magnetic performances of the alloys, to be explained hereafter.

By MAUD fitting, we were able to quantify the relative abundance of the constituent structural phases for every investigated sample. Table 3 shows the lattice parameters as well as relative abundance of the constituent phases, in the case of the P4 as-cast and annealed samples.

We can now conclusively draw the schematics of the phase evolution with the annealing temperatures, at 400 °C–500 °C, for the two ranges of composition in the MnGa binary alloy, in the range of Mn content between 70 and 75 at%.

(A) For 70 to 71 at% Mn: Hexagonal D0_19_ Mn_3_Ga → Tetragonal D0_22_ Mn_3_Ga + Tetragonal L1_0_ Mn_2_Ga + Hexagonal D0_19_ Mn_3_Ga
(B) For 73 to 75 at% Mn: Cubic Mn_3_Ga → Tetragonal D0_22_ Mn_3_Ga + Tetragonal L1_0_ Mn_2_Ga + (residual) Cubic Mn_3_Ga

It can be seen that the phase structure of the MnGa alloy is highly sensitive to small changes in stoichiometry, especially in the vicinity of the 75:25 Mn to Ga ratio, where, depending on tiny differences in the Mn to Ga ratio, either cubic or hexagonal Mn_3_Ga phases occur in the as-cast state.

Moreover, irrespective of their as-cast state, the MnGa alloys stabilize towards a mixture of tetragonal D0_22_ and L1_0_ phases, upon annealing at not so high temperatures (400 °C–500 °C). The difference is that, when departing from cubic precursor upon annealing, the relative abundance of the tetragonal phases is much higher than the content of tetragonal phases in the case the precursor is the hexagonal D0_19_ Mn_3_Ga phase. This may be due to the higher stability of the hexagonal phase, as revealed also in the MnGa phase diagram [20].

Therefore, it appears that for higher magnetic performances, having established a composition which allows formation of cubic Mn_3_Ga (instead of the more stable hexagonal structure) can be more beneficial, leading to higher amounts of ferromagnetic, tetragonal MnGa phases, such as the L1_0_ Mn_2_Ga and D0_22_ Mn_3_Ga.

This result appears extremely important since the magnetism of these alloys strongly depends on the type of structure of the alloy in a given state of annealing.

These structural results are confirmed by some direct determinations of the microstructure of the MnGa alloys by electron microscopy analysis.

### 3.2. High-Resolution TEM

**Figure 7:**
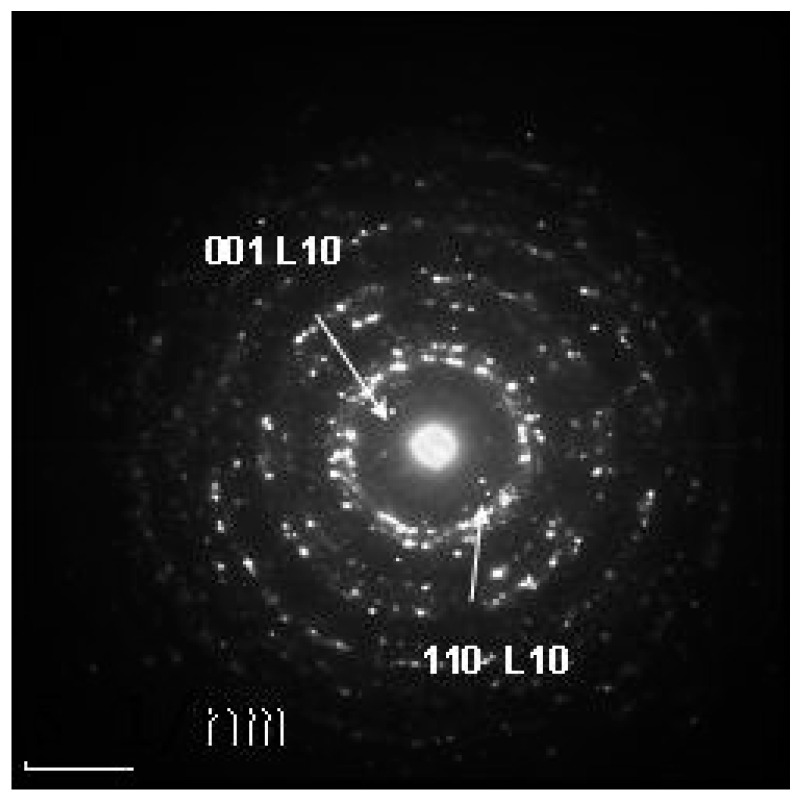
Electron diffraction pattern of sample P4 annealed at 400 °C.

**Figure 8:**
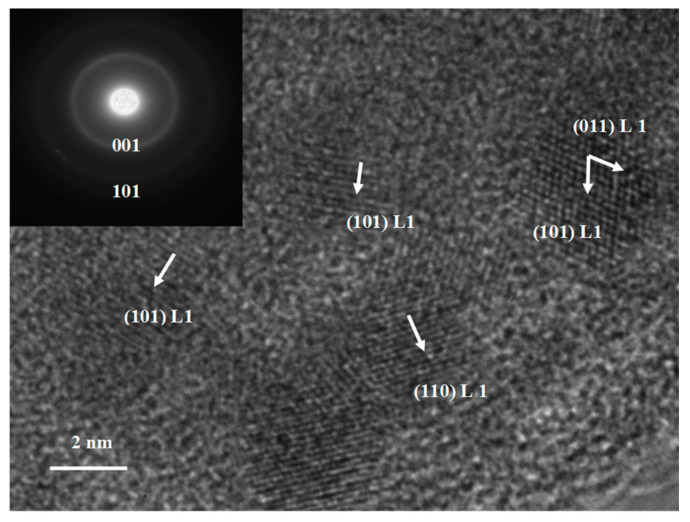
High-resolution TEM image of the sample P4 annealed at 500 °C. The inset is showing the corresponding electron diffraction pattern of the TEM image with lines belonging to the L1_0_ Mn_2_Ga phase.

Structural analysis by XRD has been complemented by TEM and EDP determinations. While the phase evolution from the as-cast state towards obtaining magnetically relevant tetragonal phases was thoroughly investigated in the previous section, we have employed TEM imaging characterization combined with EDP only on selected samples. For that purpose, we have chosen to investigate the P4 annealed samples where it was found by full-profile analysis that tetragonal phases are co-existing in the microstructure upon annealing and their abundance is the largest, among all other investigated samples. For the P4 sample annealed at 400 °C, we have obtained and present here (Figure 7) the electron diffraction pattern, as a map of the diffraction pattern in the reciprocal space. Here, we can see that there are bright spots organized in rings which corresponds to *hkl* reflections in the direct space. By measuring the distances from the center of the image to the bright spots, we have been able to calculate the reticular distances and we could assign unambiguously the coherent rings to both the (001) and (110) superlattice peaks which belong to the tetragonal L1_0_ Mn_2_Ga phase. Figure 8 depicts an example of a high-resolution TEM image of the P4 sample annealed at 500 °C, as well as its selected area electron diffraction pattern, as inset. Here, we can see that the samples exhibit a nanogranular microstructure, with nanometric-sized regions. Nanometric-sized small crystals are observed and imaged in high resolution. These small, observed nanocrystals have 3 to 6 nanometers, in average size, and are dispersed into a residual matrix of a different nature, as revealed by the difference in contrast as well as in the crystallinity of the surrounding region. The observation allowed us to detect and image neatly the parallel atomic planes where we could measure the *d*-spacings between these planes.

The results are in very good agreement with some of the measured Bragg reflections which belong to the tetragonal L1_0_ phase. Therefore, we have identified and confirmed with this the XRD results, which showed that the superlattice peaks, attributed to the (001), (011) and (101) reflections of the L1_0_ phase, are present in the P4 sample, annealed at 500 °C. As inset, the selected area electron diffraction taken from the same area imaged by high resolution TEM, exhibit two less pronounced diffraction rings. By measuring their distance to the center of the image, we have unambiguously determined their attribution to the (001) and (101) reflections of the tetragonal L1_0_ Mn_3_Ga phase, respectively. By adding this complementary information, we have furthermore proven that the phase structure of the P4 sample annealed at 500 °C is dominated by the tetragonal L1_0_ phase, in addition to the tetragonal D0_22_, detected in XRD. Both these phases are of strong interest for developing the MnGa system as a further class of permanent magnets.

### 3.3. Magnetic Characterization

A detailed set of magnetic measurements were performed on the samples, in both their as-cast and annealed state, in order to complete the characterization and to clarify the influence of the phase structure observed in structural measurements on the overall magnetic performances of the samples. For that purpose, we have made use of the vibrating sample magnetometry module of our MPMS facility. In the beginning of the study, the protocol of the measurement foresaw first the fixation on the sample holder and then centering the sample in the spot where an optimal magnetic moment is found within the measurement coil. Afterwards, the initial magnetization of the mounted samples was recorded, without the sample having its magnetic moment oriented by applying a magnetic field. This is performed in order to keep the pristine nature of the samples, allowing us to accurately measure magnetic moments in the applied magnetic field, without undesired previous magnetization history. Each of the VSM measurements was recorded on ribbons cut to fit into roughly 2 mm × 2 mm × 0.04 mm. The ribbon piece was mounted on the holder, and we afterwards applied a variable magnetic field in a direction which was kept parallel to the ribbon’s plane. The weight of the ribbon piece measured was about 1.36 mg. Demagnetization factor of the sample was calculated assuming the size of the ribbon piece measured (2 mm × 2 mm × 0.04 mm) to be around 1.5 × 10^−4^, following the model developed for magnetic melt-spun ribbons by D.N. Zhmetko [38]. Corrections for the demagnetization factor were carefully applied before plotting the magnetization results.

The ultimate purpose of this study is to determine the magnetic behavior in terms of coercivity and saturation magnetization of the annealed samples, as the annealed samples are the ones where the tetragonal, hard magnetic phases are found to co-exist in the four different compositions of MnGa binary alloy. Moreover, both coercivity and saturation magnetization are to be taken into consideration when assessing the figure of merit of any permanent magnet, as they constitute the basis of calculus for the maximum energy product (BH) max, or in other words the maximum magnetic energy that can be stored by each piece of magnet, used in any industrial applications.

**Figure 9:**
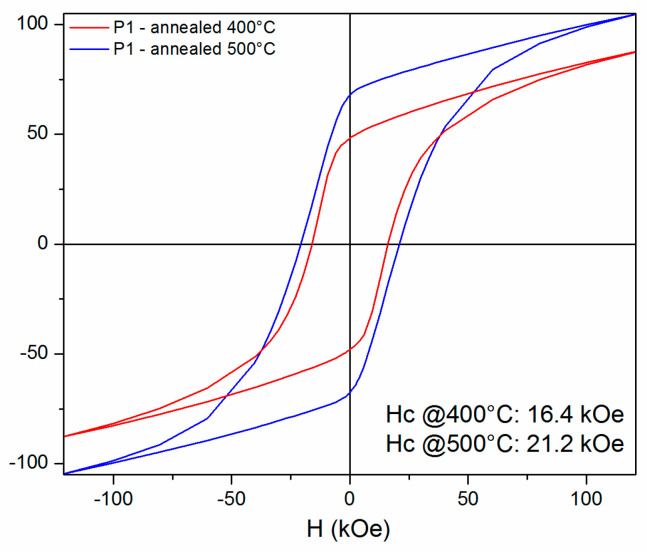
The 300 K hysteresis loops recorded under an applied field of up to 12 Tesla, for the P1 samples annealed at 400 °C and 500 °C. The field is applied parallel to the ribbons plane.

**Figure 10:**
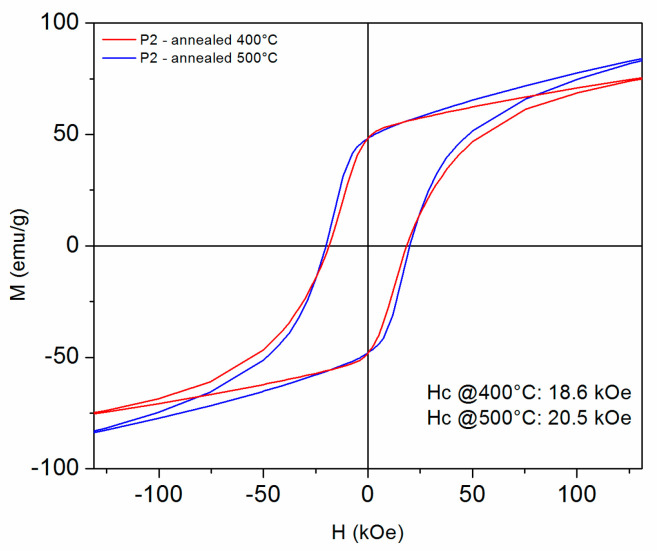
The 300 K hysteresis loops recorded under an applied field of up to 12 Tesla, for the P2 samples annealed at 400 °C and 500 °C. The field is applied parallel to the ribbons plane.

**Figure 11:**
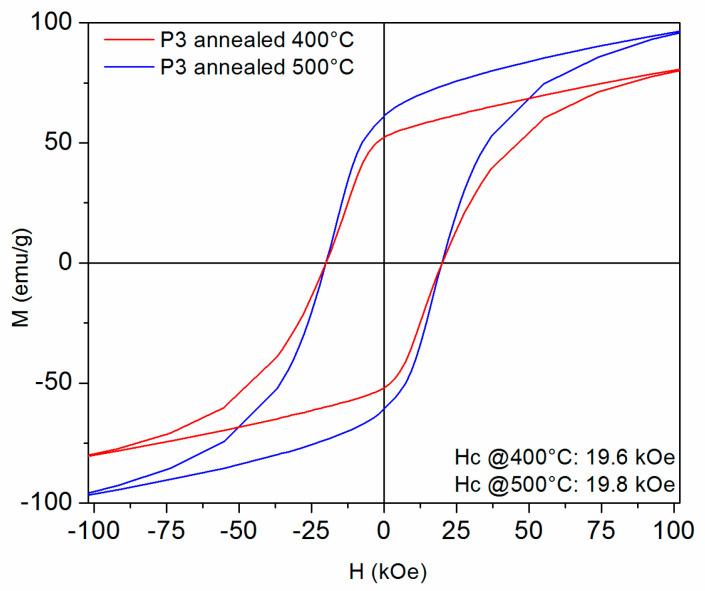
The 300 K hysteresis loops recorded under an applied field of up to 12 Tesla, for the P3 samples annealed at 400 °C and 500 °C. The field is applied parallel to the ribbons plane.

**Figure 12:**
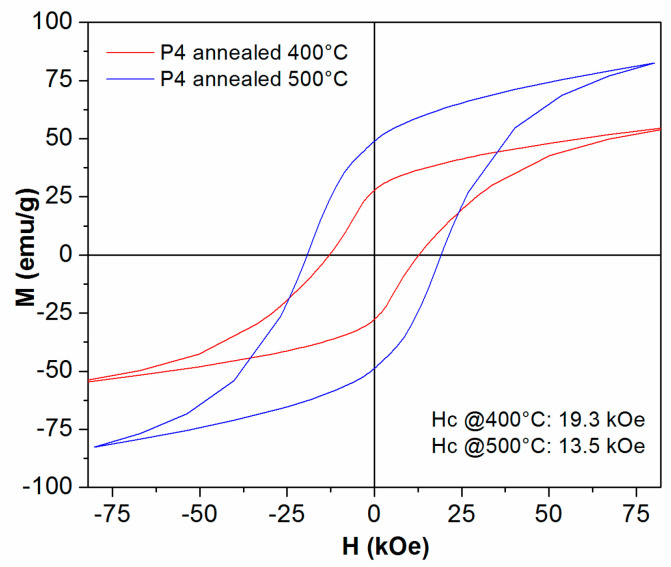
The 300 K hysteresis loops recorded under an applied field of up to 12 Tesla, for the P4 samples annealed at 400 °C and 500 °C. The field is applied parallel to the ribbons plane.

Figure 9, Figure 10, Figure 11 and Figure 12 show the 300 K hysteresis loops of the samples, from P1 up to P4, annealed at 400 °C and 500 °C.

The hysteresis loops recorded at 300 K under a magnetic field applied parallel to the ribbon plane of up to 12 Tesla were also measured for all of the four as-cast samples; however, they show very low specific magnetization (less than 5 emu/g) and also very low coercive fields (of the order of 30–50 Oe). Therefore, we have chosen not to represent them here. These low magnetization values are linked to the pronounced ferrimagnetic character in the precursor phase, for the P1 up to P4 as-cast samples. On the contrary, the two annealed P1 samples show a strong increase in both net magnetization and coercivity. Moreover, the two annealed samples show no saturation, even at the highest applied field.

The sample annealed at 400 °C exhibits a maximum specific magnetization of about 87 emu/g, while the sample annealed at 500 °C shows a larger value of the maximum specific magnetization of about 102 emu/g. Taking into account the density of the sample (6.69 g/cm^3^), we calculated the maximum magnetization to be around 0.59 M A/m for the sample annealed at 400 °C and 0.63 M A/m for the sample annealed at 500 °C, respectively.

In the case of the P2 samples, also annealed at 400 °C and 500 °C, the behavior is quite similar, but in this case, there is not much difference in terms of saturation magnetization and coercivity between the two different annealing temperatures. Here, the coercivity amounts to 18.6 and 20.5 kOe, respectively, for the two annealing temperatures. Saturation magnetization reaches 75 emu/g (0.5 M A/m) and 88 emu/g (0.59 M A/m).

The P3 samples also show quite similar behavior between the two annealing conditions, with rather similar shapes of the hysteresis, slightly larger specific magnetization for the sample annealed at 500 °C but quite close to each other coercive fields: 19.6 and 19.8 kOe, respectively. Here, the saturation magnetization reaches 79 emu/g (0.53 M A/m) and 97 emu/g (0.65 M A/m), respectively.

In the case of the P4 samples, the situation is rather different, since here, the differentiation between the annealing at 400 °C and 500 °C is more pronounced. In this case, the sample annealed at 400 °C has 13.5 kOe coercivity and 55 emu/g (0.37 M A/m) saturation magnetization. The annealing at 500 °C produces a significant increase in both parameters. It means that here, the coercive field is much larger (19.3 kOe) and specific magnetization is also increased up to 83 emu/g (0.55 M A/m). These very large values of coercivity and magnetization are consistent throughout the whole series of samples and are encouraging in view of potential applications as permanent magnets and will be discussed in the following section.

## 4. Discussion

It is widely conceived that in the MnGa binary alloys, in bulk shape, the magnetization continuously increases with increasing Ga content and decreasing Mn content (75 at% down to 66 at%) [39]. This was interpreted in the frame of the “manganese dilemma”: if a large magnetic moment is to be obtained, then the Mn atoms need to be quite well separated. But this separation, or the increasing distance between neighboring Mn atoms, at the same time decreases the magnetization [24] because the magnetization is by definition calculated as the magnetic moment per unit volume. This infers a change in the Mn site occupancy. When Mn content diminishes below 75 at%, towards the needed formula of a Mn_2_Ga phase, rather than a Mn_3_Ga one, more and more Mn atoms will migrate to the 4d sites in the tetragonal unit cell, which increases the magnetization in Mn_2_Ga compared to the Mn_3_Ga. In our case, the phase structure of the annealed samples contains both the L1_0_ Mn_2_Ga tetragonal phase as well as the Mn_3_Ga D0_22_ tetragonal phase in various proportions, and this produces these very large values of magnetization, between 0.37 and 0.68 M A/m. This is influenced, moreover, by the exchange interaction between the tetragonal nanosized grains, both intra-phase and inter-phase since there are two tetragonal phases present in the annealed samples and its strong dependence on interatomic Mn–Mn distances that mainly governs the arrangement of magnetic Mn-moments and, hence, the magnetization of the alloys. Indeed, in the paper of J.M.D. Coey [24], it has been shown that Mn atoms on sites distanced by the shortest Mn–Mn distances, <240 pm, tend to be nonmagnetic, the Mn atoms on sites distanced between 250 and 280 pm tend to couple antiferromagnetically and only Mn atoms on sites with the longest bonds, >290 nm, tend to have larger moments, ferromagnetically coupled. The values we obtained (maximum 0.68) are very much close to the maximum predicted value of 0.7 M A/m in Mn_2_Ga if all Mn atoms would occupy the *4d* sites of the tetragonal unit cell, retaining their moment of 2.08 µB [24]. This constitutes a very encouraging result and a significant increase compared with other reported coercive field values in the literature. Most of our coercive field values are anyway higher than the values of about 0.47 M A/m encountered in thin films of Mn_3_Ga [40]. This result is in agreement with the suggestion formulated in [24] that there is room for improvement and optimization of magnetic performances around areas with D0_22_ structure or in the regions in the Mn–Ga phase diagram where the L1_0_ phase appears. Similarly good results in systems involving D0_22_ phase were also obtained in the case of the MnGe system [41,42]

The most remarkable results are represented by the very large values of the coercive field and of the remanence shown for all of our two annealed samples. The values are consistent also throughout the very large range of composition from 70 at% up to 75 at% Mn content. These values exceed the results obtained in MnGa thin films by far, as well as in other MnGa bulk alloys, with lower Mn content, reported in the literature, and the highest value encountered in the literature for the MnGa binary system being 13.5 kOe [25]. For comparison, we can provide several other values for Mn-Ga systems, thin films, layers and bulk alloys, from the literature. For instance, in [43] it was shown that L1_0_ MnGa thin films grown on an amorphous glass substrate, show increase in magnetization and coercivity, upon increasing the thickness, with maximum *M*_10kOe_ and *H*_c⊥_ values (106.3 ± 5.7 emu/cm^3^ and 8.54 kOe) attained in a 200 nm thick sample. In [44], L1_0_-Mn_1.60_Ga thin films were prepared on MgO (100) substrates by magnetron sputtering under different substrate temperatures ranging from 200 to 600 °C, with optimal coercivity of 7.59 kOe and effective anisotropy of film being encountered at 400 °C substrate temperature. In [45], upon growing MnGa thin films with a perpendicular magnetic anisotropy on the BiSb topological insulator, both the L1_0_ phase (x < 0.6) and the D0_22_ phase (x > 0.6) of Mn*x*Ga_1−*x*_ were found, having 12 kOe coercivity. For 0.50 ≤ x ≤ 0.55, ferromagnetic single-phase L1_0_-MnGa thin films were detected. In [46], a pseudo-morphic deposition of L1_0_ MnGa nanolayers at room temperature on B2-ordered paramagnetic CoGa templates yielded good perpendicular magnetic anisotropy and a coercive field of about 8 kOe.

These high coercive values can be attributed to the exchange coupling between the two tetragonal phases present in the samples, as well as the coupling between the tetragonal nanograins. It is inferred that the competing effect of the D0_22_ magnetic sublattice exchange coupled with the magnetic moments of small nanocrystals of tetragonal L1_0_ phases might give these large values of remanence and coercivity. However, these findings need further and deeper investigations.

## 5. Conclusions

We successfully produced four different MnGa magnetic alloys via an out-of-equilibrium method of ultra-fast solidification from the melt, and we succeeded in stabilizing the co-existence of two different tetragonal phases: the D0_22_ Mn_3_Ga and L1_0_ Mn_2_Ga, by isothermal annealing at 400 °C and 500 °C of the rapidly solidified ribbons. Extended structural analysis by XRD, TEM imaging and EDP showed that the MnGa alloys submitted to thermal annealing in optimal conditions has a microstructure, where small nanocrystals of tetragonal L1_0_ co-exist with the tetragonal D0_22_ phase, in various content ratios. Depending on the Mn content, we identify the schematics of structural phase transformation upon annealing, as follows: (a) For 70 to 71 at.%Mn: Hexagonal D0_19_ Mn_3_Ga → Tetragonal D0_22_ Mn_3_Ga + Tetragonal L1_0_ Mn_2_Ga + Hexagonal D0_19_ Mn_3_Ga; (b) For 73 to 75 at.%Mn: Cubic Mn_3_Ga → Tetragonal D0_22_ Mn_3_Ga + Tetragonal L1_0_ Mn_2_Ga + (residual) Cubic Mn_3_Ga. These co-existing, hard magnetic phases produce an optimal set of promising magnetic properties, with very a high coercive field accompanied by a large enough remanence, higher than the values reported in the literature for single-phase MnGa alloys and thin films. Such large values are explained by the exchange coupling between magnetic sublattices of the D0_22_ Mn_3_Ga with the net moment of the tetragonal L1_0_ Mn_2_Ga nanocrystals. These findings are extremely promising in view of potential applications of the MnGa binary system as a promising novel permanent magnet material which avoids the use of rare earths.

## Figures and Tables

**Table 1:** Nominal compositions and real compositions, measured via EDX.

Sample	Nominal Composition	EDX Compositions ^1^
Sample P1	Mn_70_Ga_30_	Mn_69.6_Ga_30.4_
Sample P2	Mn_71_Ga_29_	Mn_70.7_Ga_29.3_
Sample P3	Mn_73_Ga_27_	Mn_72.6_Ga_27.4_
Sample P4	Mn_75_Ga_25_	Mn_74.4_Ga_25.6_

^1^ Compositions are given in atomic percent.

**Table 2:** Lattice parameters for the two tetragonal phases and the hexagonal one, as resulted from the MAUD fitting for the P2 samples.

Sample	D0_19_	D0_22_	L1_0_	Abundance
P2 annealed at 400 °C	*a* = 2.689 Å *c* = 4.348 Å	*a* = 3.861 Å *c* = 7.072 Å	*a* = 3.869 Å *c* = 3.566 Å	D0_19_: 47% D0_22_: 20% L1_0_: 33%
P2 annealed at 500 °C	*a* = 2.678 Å *c* = 4.314 Å	*a* = 3.854 Å *c* = 7.061 Å	*a* = 3.862 Å *c* = 3.558 Å	D0_19_: 41% D0_22_: 22% L1_0_: 37%

Errors in the lattice parameters determination: ±0.003 Å to ±0.007 Å. Errors in the abundance determination: ±1.2% to ±2.1%.

**Table 3:** Lattice parameters for the two tetragonal phases and the cubic one, as resulted from the MAUD fitting for the P4 samples.

Sample	Cubic	D0_22_	L1_0_	Abundance
P4 annealed at 400 °C	*a* = 3.772 Å	*a* = 3.852 Å *c* = 7.054 Å	*a* = 3.871 Å *c* = 3.533 Å	cubic: 3% D0_22_: 42% L1_0_: 55%
P4 annealed at 500 °C	*a* = 3.785 Å	*a* = 3.848 Å *c* = 7.047 Å	*a* = 3.867 Å *c* = 3.541 Å	cubic: 2% D0_22_: 39% L1_0_: 59%

Errors in the lattice parameters determination: ±0.006 Å to ±0.011 Å. Errors in the abundance determination: ±0.7% to ±1.4%.

## Data Availability

The data presented in this study are available on request from the corresponding author. The data are not publicly available due to IPR protection measures.
